# Genes Associated With Fracture Risk in Thoroughbred Horses Have Novel Roles in Osteogenesis

**DOI:** 10.1002/age.70166

**Published:** 2026-07-08

**Authors:** Amy C. Ross, Ellison S. Lumsden, Caroline Flood, Jayesh Dudhia, Androniki Psifidi, Deborah J. Guest

**Affiliations:** ^1^ Department of Clinical Sciences and Services, Centre for Vaccinology and Regenerative Medicine The Royal Veterinary College Hatfield Herts UK

**Keywords:** bone, fracture, genetics, horse, mineralisation, osteoblast, osteogenesis

## Abstract

Bone fractures in Thoroughbred racehorses are a major welfare problem. Genetic factors contribute to fracture risk. Cell models have previously identified 112 differentially expressed genes in bone‐forming osteoblasts derived from horses at high and low genetic risk of fracture. However, 42 of these genes have no published role in bone. In this study, we identified novel roles for a subset of these genes in bone formation. Twenty‐six of the 42 genes were expressed in Saos2 cells during basal culture and/or after 21 days of osteogenic culture. Five of these genes (*ADSSL1*, *CABP1*, *ENO2*, *SPARCL1* and *UCP2*) were then stably overexpressed and knocked down, and their effect on osteogenesis was measured. Gene overexpression resulted in significant decreases in Saos2 cell viability and decreased expression of osteogenic genes under basal cell culture, but after 21 days of osteogenic culture there were few significant changes in osteogenic gene expression, collagen deposition or matrix mineralisation. Knockdown of *SPARCL1* resulted in total cell death, whereas knockdown of *ADSSL1*, *CABP1*, *ENO2* and *UCP2* resulted in decreased cell viability but limited significant changes in osteogenic gene expression under basal cell culture. However, following osteogenic culture, gene knockdown induced widespread changes in osteogenic gene expression, decreased collagen deposition and increased matrix mineralisation. *ADSSL1*, *CABP1*, *ENO2* and *UCP2* were all expressed at significantly lower levels in osteoblasts from genetically high‐risk horses. Taken together, this work demonstrates novel roles for fracture‐associated genes in bone formation and matrix mineralisation suggesting these processes may be altered in genetically susceptible horses.

## 1 Introduction

Bone fractures commonly occur in Thoroughbred racehorses and can result in euthanasia. The global incidence of such catastrophic fractures is around 1 per 1000 horse starts (Wright et al. 2024), but this can vary depending on the jurisdiction, race type, track surface and reporting criteria (Wright et al. 2024). Fracture is the leading cause of euthanasia on the racecourse (Wright et al. 2024) accounting for approximately 75% of all racecourse fatalities (Rosanowski et al. 2018). Fracture therefore has a significant economic and welfare impact.

A large proportion of bone fractures are believed to arise from a failure of the bone to adapt to normal loading and thus may be preventable (Riggs 2002). Many factors underpin fracture risk and include both environmental (Georgopoulos and Parkin 2017) and genetic factors (Blott et al. 2014; Tozaki et al. 2020), with heritability estimates of 0.21–0.37 (Welsh et al. 2014; Tozaki et al. 2019). We have previously developed a genome‐wide polygenic risk score (PRS) for catastrophic fracture in Thoroughbreds (Palomino Lago et al. 2024) and used this to establish cell models to study osteoblasts from genetically high‐ and low‐risk horses (Palomino Lago et al. 2024, 2025).

Using these models we identified 112 genes that are significantly differentially expressed in iPSC (induced pluripotent stem cell)‐osteoblasts derived from Thoroughbred horses at high and low genetic risk of fracture (Palomino Lago et al. 2025). The differentially expressed genes were overrepresented in biological processes regulating the extracellular matrix and pathways involved in bone remodelling. However, of the 112 differentially expressed genes, 27 were not annotated and, of the remaining 85 genes, only 43 have published roles in bone formation and/or fracture (Palomino Lago et al. 2025). The remaining 42 annotated, differentially expressed genes have no published role in bone. Nevertheless, of these genes, 37 are reported to be expressed in equine bone tissue (Kuemmerle et al. 2016; Kemper et al. 2019). Furthermore, we demonstrated that 18 of the genes encoded proteins which interacted (directly or indirectly) with proteins which have known roles in bone and are also encoded by differentially expressed genes (Palomino Lago et al. 2025). We therefore hypothesised that many of these genes have roles in bone that had not yet been discovered. In this study, we aimed to determine if some of these genes had novel roles in bone formation.

Studying gene function requires the use of cells that are accessible, easy and cost‐effective to culture, amenable to genetic manipulation and can undergo significant expansion in culture. At the time of this study, suitable equine bone cell lines were not available. However, we had previously demonstrated that the human osteosarcoma cell line, Saos2, represents a mature osteoblast‐like cell that has the capacity to robustly generate a mineralised matrix following osteogenic culture and can be used to study gene function (Ross et al. 2026).

We first determined the expression of the 42 genes of interest in Saos2 cells during osteogenic culture and then prioritised five candidate genes to take forward for modulation to determine their roles during osteogenesis by Saos2 cells. Understanding the function of genes associated with fracture risk is a critical step for the future development of new interventions for genetically high‐risk horses.

## 2 Materials and Methods

**FIGURE 1:**
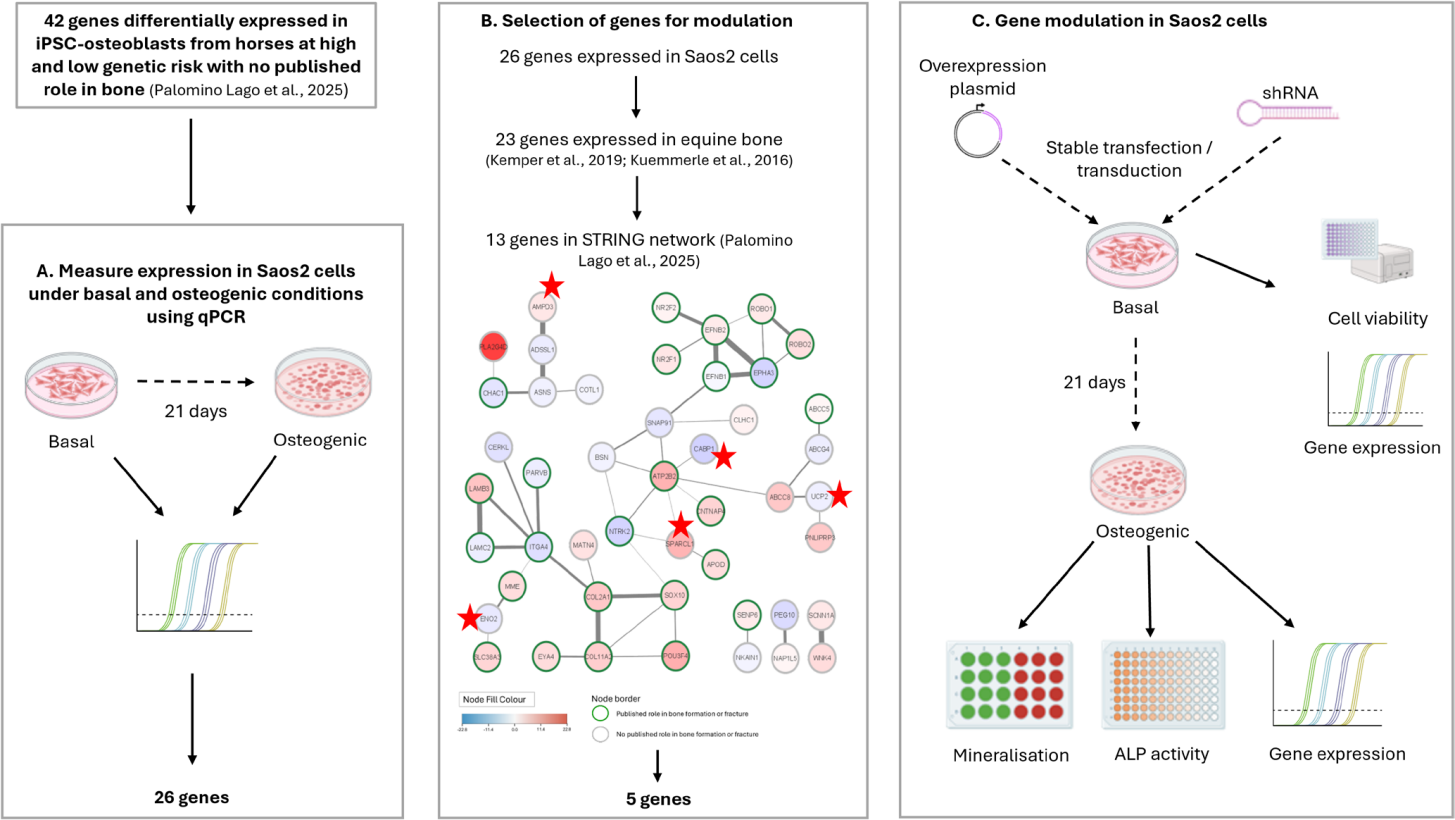
Experimental overview. Red stars indicate the genes investigated in this study.

The methods used in this study are all based on our previously published methods on gene modulation in Saos2 cells (Ross et al. 2026). An overview of our experimental approach is provided in Figure 1.

### 2.1 Saos2 Cell Culture

For basal, proliferative culture, Saos2 cells (RRID: CVCL_0548; HTB‐85 from ATTC, USA) were grown in basal growth media consisting of McCoys 5A (high glucose [3000 mg/L] without sodium pyruvate but with L‐glutamine [219.2 mg/L]), with the addition of 15% foetal bovine serum (FBS), 1000 U/mL penicillin and 100 μg/mL streptomycin (P/S) (All Thermo Fisher, USA). Cells were passaged with 0.25% trypsin–EDTA upon reaching 70%–80%.

For osteogenic culture, the Saos2 cells were grown to 70%–80% confluency and then the media changed to osteogenic media (basal media supplemented with 10 nM dexamethasone, 280 μM ascorbic acid and 5 mM β‐glycerophosphate [All Sigma, UK]). Osteogenic media was changed every 3–4 days for 21 days prior to analyses.

### 2.2 Gene Overexpression

Saos2 cells were seeded in six‐well plates in basal media lacking penicillin/streptomycin and cultured until 70%–80% confluent and then transfected with 2.5 μg of pCMV6‐AC‐GFP (OriGene, UK) per well. Plasmids carrying the open reading frame of each gene of interest were used alongside an empty vector control plasmid (see Table 1). Lipofectamine 3000 (L3000) (Invitrogen) was used for the transfections according to the manufacturer's instructions. Forty‐eight hours post‐transfection, antibiotic selection was carried out with 1 mg/mL of G418 (Sigma‐Aldrich, dose optimised by kill curve on non‐transfected cells), and resistant cells were expanded to create stable overexpression cell lines.

**TABLE 1:** Overexpression constructs (all from OriGene).

Gene	Accession number	Product number
*ADSSL1*	NM_152328	RG208536
*CABP1*	NM_031205	RG218317
*ENO2*	NM_001975	RG201085
*SPARCL1*	NM_004684	RG207583
*UCP2*	NM_003355	RG203997
Empty vector control	—	PS100010

### 2.3 Gene Knockdown

Lentiviral vectors carrying commercially available shRNAs were used to generate Saos2 cells with stable knockdowns of the genes of interest. Briefly, HEK293T packaging cells (RRID: CVCL_0063) were seeded onto a six‐well plate at a density of 1 × 10^5^ cells per well in Dulbecco's modified Eagle's medium (DMEM; high glucose [4500 mg/L] with sodium pyruvate [110 mg/L]), 10% foetal bovine serum (FBS) and 2 mM L‐glutamine (LQ) (all Gibco, Thermo Fisher Scientific). Twenty‐four hours post‐seeding, each well was transfected with a combination of 1 μg lentiviral TRC1‐pLK0.1 plasmid carrying either a specific shRNA sequence for the genes of interest (see Table 2) or a non‐target (NT) scrambled shRNA (5′‐GCGATAGCGCTAATAATTT‐3′ SHC202; Sigma‐Aldrich), along with 750 ng psPAX2 and 250 ng pMD2.G using FuGENE 6 transfection reagent (Promega, UK) according to the manufacturer's instructions. pMD2.G and psPAX2 were a gift from Didier Trono (Addgene plasmids #12259 and 12260; http://n2t.net/addgene:12259 http://n2t.net/addgene:12260; RRID: Addgene_12259 and 12260). As positive control to visualise transfection and transduction, TRC2‐pLKO.5‐puro‐CMV‐TurboGFP plasmid (SHC 203; Sigma‐Aldrich) was used.

**TABLE 2:** shRNA sequences used for knockdown (all from Sigma‐Aldrich). Two different sequences were tested for SPARCL1.

Target gene	Target sequence (5′–3′)	Product number
*ADSSL1*	CCGAGCAGATCAACGAGATTG	TRCN0000232446
*CABP1*	CGAGATGCTTTCCGAGAGTTT	TRCN0000029359
*ENO2*	CAAGGGAGTCATCAAGGACAA	TRCN0000157687
*SPARCL1* (design A)	ATACCCAATCTGATGATATTT	TRCN0000373631
*SPARCL1* (design B)	CCCGACAAATGCAAGATTATT	TRCN0000055755
*UCP2*	CGTGGTCAAGACGAGATACAT	TRCN0000060144

At 24 h post‐transfection, the media was changed to Saos2 cell basal growth media (without penicillin/streptomycin), and 48 h later, packaging cell supernatant was harvested, filtered through a 0.45 μm filter (Millipore, Billerica, USA), combined with 10 μg/mL polybrene (Sigma‐Aldrich) and immediately applied to Saos2 cells, which were plated at a density of 2 × 10^5^ cells per well 24 h before transduction. One round of viral infection was carried out for 24 h, which resulted in ~80% of the cells being transduced. Seventy‐two hours after the transduction, media containing 2.5 μg/mL puromycin (Sigma‐Aldrich, dose selected using a kill curve on non‐transduced cells) was used to select stably transduced cells.

### 2.4 Cell Viability

Saos2 cells were seeded in 96‐well plates at a density of 1 × 10^4^ cells per well in basal media. Following 48 h incubation at 37°C and 5% CO_2_, culture media was removed and 100 μL of diluted PrestoBlue reagent (1:10; Invitrogen, Thermo Fisher) was added to each well and the plates were incubated at 37°C for 10 min. Fluorescence for individual wells was measured using a Tecan plate reader (TECAN infinite M nano+) at 560 nm/590 nm excitation and emission wavelengths respectively. Viability was measured in seven technical replicates and is shown in relative fluorescence units (RFU) as a measure of the amount of resorufin (produced from the reduction of resazurin by viable cells).

### 2.5 RNA Extraction, cDNA Synthesis, and qPCR

Cells were harvested into Tri‐Reagent (Sigma) and the Qiagen RNAeasy kit (Qiagen, Hilden, Germany) was used to extract RNA. Genomic DNA was removed using the Invitrogen DNA‐free DNA Removal Kit (Thermo Fisher). RNA concentrations were calculated using a Nanodrop 1000 spectrophotometer (Thermo Fisher) and 260:280 ratios were confirmed to be approximately 2.0. For cDNA synthesis, 1 μg of RNA was reverse transcribed using the SensiFAST cDNA synthesis kit (Bioline, London, UK) on a Bio‐Rad T100 Thermal Cycler (Bio‐Rad, Hertfordshire, UK) according to the manufacturer's instructions. For qPCR, 2 μL of cDNA (20 ng) was used per reaction with SensiMix SYBR Green supermix (Bioline) on a Bio‐Rad C1000 Touch Thermal Cycler (Bio‐Rad). Reactions were performed in duplicate. qPCR cycle parameters were 95°C for 10 min followed by 45 cycles of 95°C (15 s), 60°C (15 s) and 72°C (15 s). This was followed by a melt curve with readings taken every 1°C from 65°C to 95°C. Relative gene expression to the *ACTB* housekeeping gene was calculated using 2^−∆CT^ (Livak and Schmittgen 2001). *ACTB* stability had been previously confirmed (Ross et al. 2026). Primers to human genes were designed as described previously (Ross et al. 2026) and their sequences can be found in Table S1. Gene expression was measured in three to five independent, experimental replicates (where an experimental replicate represents cells at different passages set up and harvested on different dates to allow the consistency of the effects across different experimental runs to be determined; Lazic et al. 2018). Gene expression following gene modulation was summarised in heatmaps created in GraphPad Prism version 10.1.2(324) using *z*‐score normalisation across either all basal gene expression data or all osteogenic gene expression data.

### 2.6 Mineralisation Assays

Saos2 cells were cultured in 24‐well plates under osteogenic culture conditions for 21 days. Cells were fixed using 3% paraformaldehyde for 20 min at room temperature and then stained for calcium deposition using 2% Alizarin Red S, pH 4.2 (Sigma), for 5 min at room temperature. For the detection of hydroxyapatite, cells were stained using the OsteoImage kit (Lonza, Slough, UK) following the manufacturer's instructions. For the detection of collagen, fixed cells were stained using a Picrosirius Red stain kit (Abcam, ab150681). Briefly, the fixed cells were hydrated with distilled water and then stained with picrosirius red solution for 1 h at room temperature. Images were captured on an EVOS XL core or EVOS FL (ThermoFisher) across a minimum of three independent, experimental replicates and analysed using ImageJ (National Institutes of Health, USA) to quantify the percentage coverage of positive staining.

### 2.7 Statistical Analysis

Statistical analysis was performed using GraphPad Prism (version 10.1.2(324)). All data sets were examined for Gaussian distribution using the Shapiro–Wilks normality test and equal variance using the *F*‐test (two groups) or Brown‐Forsythe test (more than two groups). For normally distributed data with two groups an unpaired Student's *t*‐test was used. If equal variance could not be assumed an unpaired *t*‐test with Welch's correction was performed, and if data were not normally distributed a Mann–Whitney *U* test was used. For normally distributed data with more than two groups, a one‐way ANOVA with Dunnett's post hoc test was used. If equal variance could not be assumed a Brown‐Forsythe ANOVA with Games‐Howell post hoc test was performed. If data were not normally distributed, a Kruskal–Wallis with Dunn's post hoc test was used. Significance is indicated as *p* < 0.05.

## 3 Results

### 3.1 A Large Proportion of Differentially Expressed Genes With No Published Role in Bone Are Expressed by Saos2 Cells

To select candidate genes to investigate in this study, we first measured their expression in Saos2 cells cultured under basal and osteogenic conditions (Figure 1A). Of the 42 genes that were differentially expressed in iPSC‐osteoblasts from horses at high and low genetic risk of fracture, and that had no previously published roles in bone (Palomino Lago et al. 2025), 16 of the genes were not expressed at detectable levels in Saos2 cells in basal culture or following 21 days of osteogenic culture (*AB13*, *GPHA4*, *MATN4*, *SCNN1A*, *PCDH9*, *MUC4*, *SPRED3*, *ABCC8*, *CERKL, ADGRB1, BPIFC, BSN, NKAIN1, PLA2G4D, PNLIPRP3* and *SNAP91*) (data not shown).

**FIGURE 2:**
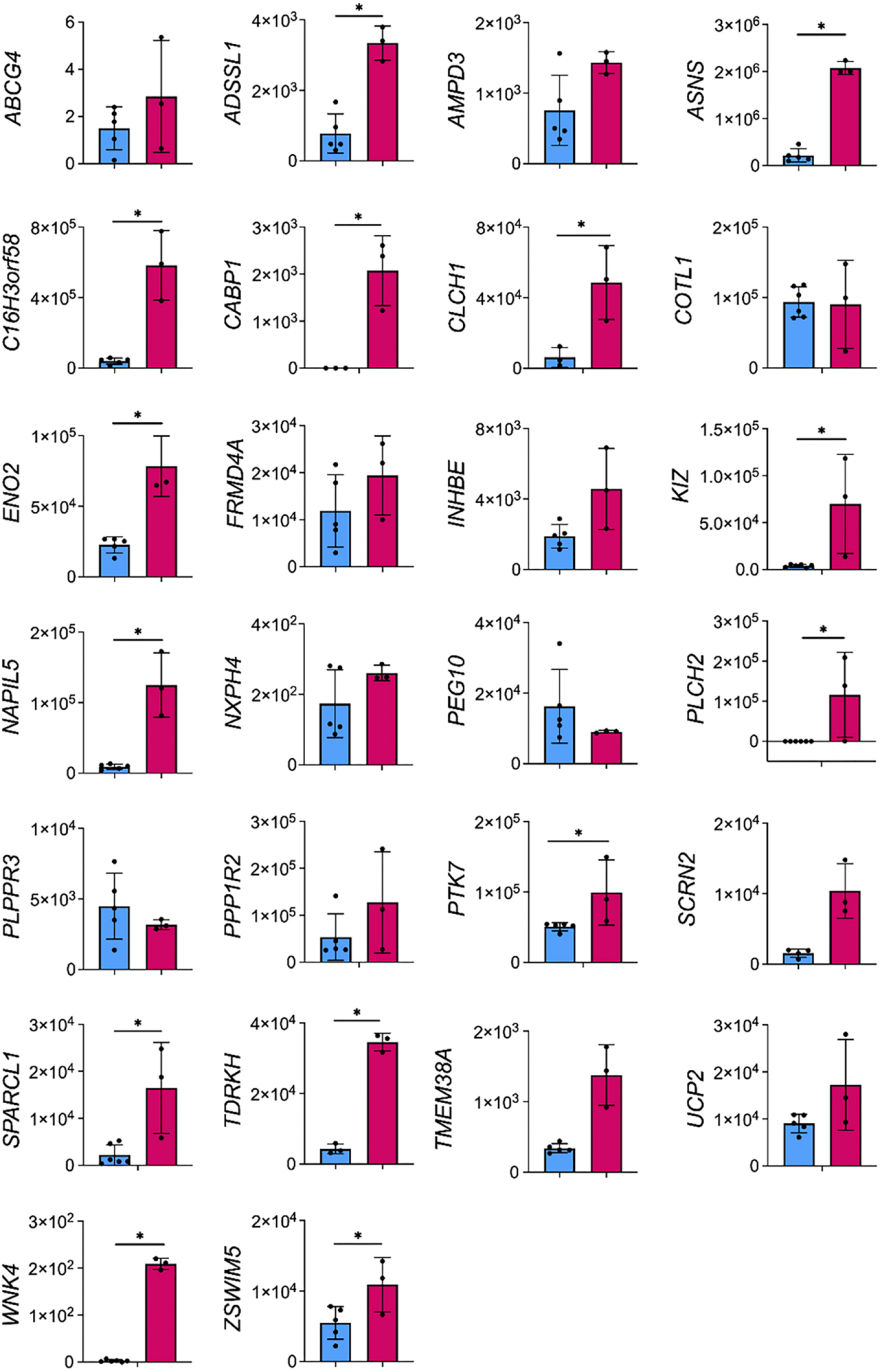
Expression of differentially expressed genes (DEGs) with no published role in bone in Saos2 cells cultured under basal and osteogenic conditions. Blue bars represent the basal conditions, and pink bars represent osteogenic conditions. Expression is shown relative to the *ACTB* housekeeping gene. Error bars represent the standard deviation from three to five experimental replicates. *p < 0.05.

Of the remaining 26 genes, 12 (*ABCG4*, *AMPD3*, *COTL1*, *FRMD4A*, *INHBE*, *NXPH4*, *PEG10*, *PLPPR3*, *PPP1R2, SCRN, TMEM38A* and *UCP2*) were expressed under both basal culture and after 21 days of osteogenic culture, with no significant changes in expression. Fourteen genes (*ADSSL1*, *ASNS*, *C16H3orf58* [also known as *DIPK2A*], *CABP1*, *CLCH1*, *ENO2*, *KIZ*, *NAPIL5*, *PLCH2*, *PTK7*, *SPARCL1*, *TDRKH*, *WNK4* and *ZSWIM5*) demonstrated a statistically significant increase in expression following osteogenic culture (Figure 2).

We then selected five candidate genes for further study in Saos2 cells (Figure 1B). Only genes which were expressed in the Saos2 cells and in equine bone tissue samples (Kuemmerle et al. 2016; Kemper et al. 2019) were included, and they were further prioritised based on the interaction of their encoded proteins with other proteins which had a published role in bone and whose encoding genes were differentially expressed in high‐ and low‐risk osteoblasts (Palomino Lago et al. 2025) and the availability of validated constructs for gene modulation. This resulted in the selection of *ADSSL1*, *CABP1*, *ENO2*, *SPARCL1* and *UCP2*. Of these genes, *ADSSL1*, *CABP1*, *ENO2* and *UCP2* were all expressed at significantly lower levels in iPSC‐osteoblasts derived from horses at genetically high risk of fracture. In contrast, *SPARCL1* was expressed at a significantly higher level in iPSC‐osteoblasts derived from high‐risk horses (Palomino Lago et al. 2025; Figure S1).

### 3.2 All Candidate Genes Could Be Overexpressed in Saos2 Cells, but SPARCL1 Was Essential for Cell Survival

**FIGURE 3:**
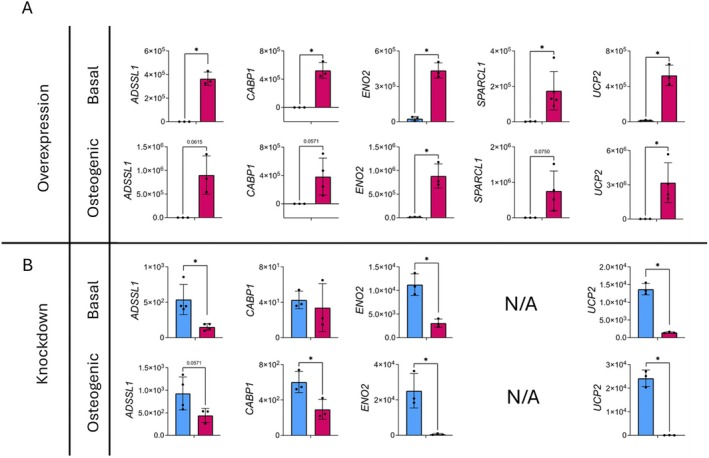
Modulation of the genes of interest. *ASSSL1*, *CABP1*, *ENO2*, *SPARCL1* and *UCP2* were modulated in Saos2 cells cultured under basal and osteogenic conditions. (A) Overexpression of the genes. Blue bars = empty vector control, pink bars = gene overexpression. (B) Knockdown of the genes. Blue bars = non‐target control, pink bars = gene knockdown. N/A = not applicable as it was not possible to isolate cells following *SPARCL1* knockdown. The error bars represent the standard deviation from 3 to 5 experimental replicates. *p < 0.05.

To determine if the five candidate genes had novel roles in osteogenesis we modulated their expression in Saos2 cells (Figure 1C). Stable transfection of a plasmid encoding the gene of interest under the control of a CMV promoter resulted in a statistically significant overexpression of all genes under basal conditions. Under osteogenic conditions, *ENO2* and *UCP2* resulted in a statistically significant overexpression. However, whilst *ADSSL1*, *CABP1* and *SPARCL1* did not reach statistical significance due to the variability in overexpression, a robust overexpression was demonstrated in all replicates (Figure 3A).

Stable expression of an shRNA to each gene of interest was then used to knockdown gene expression. *ENO2* and *UCP2* were significantly reduced under basal and osteogenic culture. Endogenous *CABP1* expression, whilst very low under basal culture, was significantly knocked down under osteogenic culture. *ADSSL1* was significantly reduced under basal culture, and under osteogenic culture a robust decrease in expression was detected but due to the variability between replicates this did not reach significance (*p* = 0.0571) (Figure 3). In contrast, it was not possible to successfully knockdown *SPARCL1*, despite testing two different shRNA sequences. In both cases, no cells survived the antibiotic selection process suggesting that *SPARCL1* is essential for Saos2 cell survival.

### 3.3 Modulation of All Genes Reduces Saos2 Cell Viability

**FIGURE 4:**
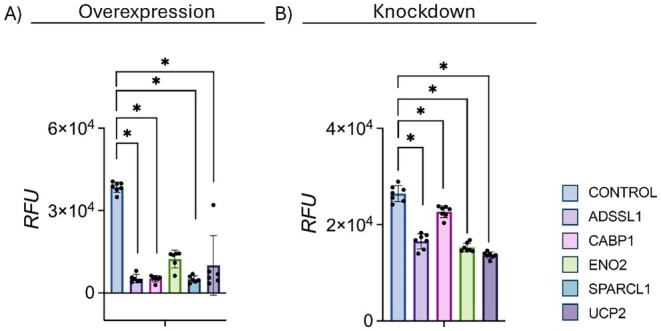
Saos2 cell viability following gene modulation. (A) Gene overexpression (control = empty vector). (B) Gene knockdown (control = non‐target). *ADSSL1* = purple bar, *CABP1 *= pink bar, *ENO2 *= green bar, *SPARCL1 *= teal bar, *UCP2 *= dark purple bar. Cell viability is shown as relative fluorescence units (RFU). Error bars represent the standard deviation from seven technical replicates. *p < 0.05.

To determine if the genes under investigation affected the cells under basal culture conditions, we first measured cell viability after the knockdown and overexpression of the genes of interest. Overexpression of all genes resulted in a large decrease in cell viability that was significantly different from the empty vector control, with the exception of *ENO2* overexpression (*p* = 0.43) (Figure 4A).

Knockdown of the genes also resulted in a significant decrease in cell viability, but to a lesser degree than following the overexpression of the genes (Figure 4B).

### 3.4 Overexpression of Candidate Genes Results in Significant Reductions in the Expression of Osteogenic Genes Under Basal but Not Osteogenic Conditions

**FIGURE 5:**
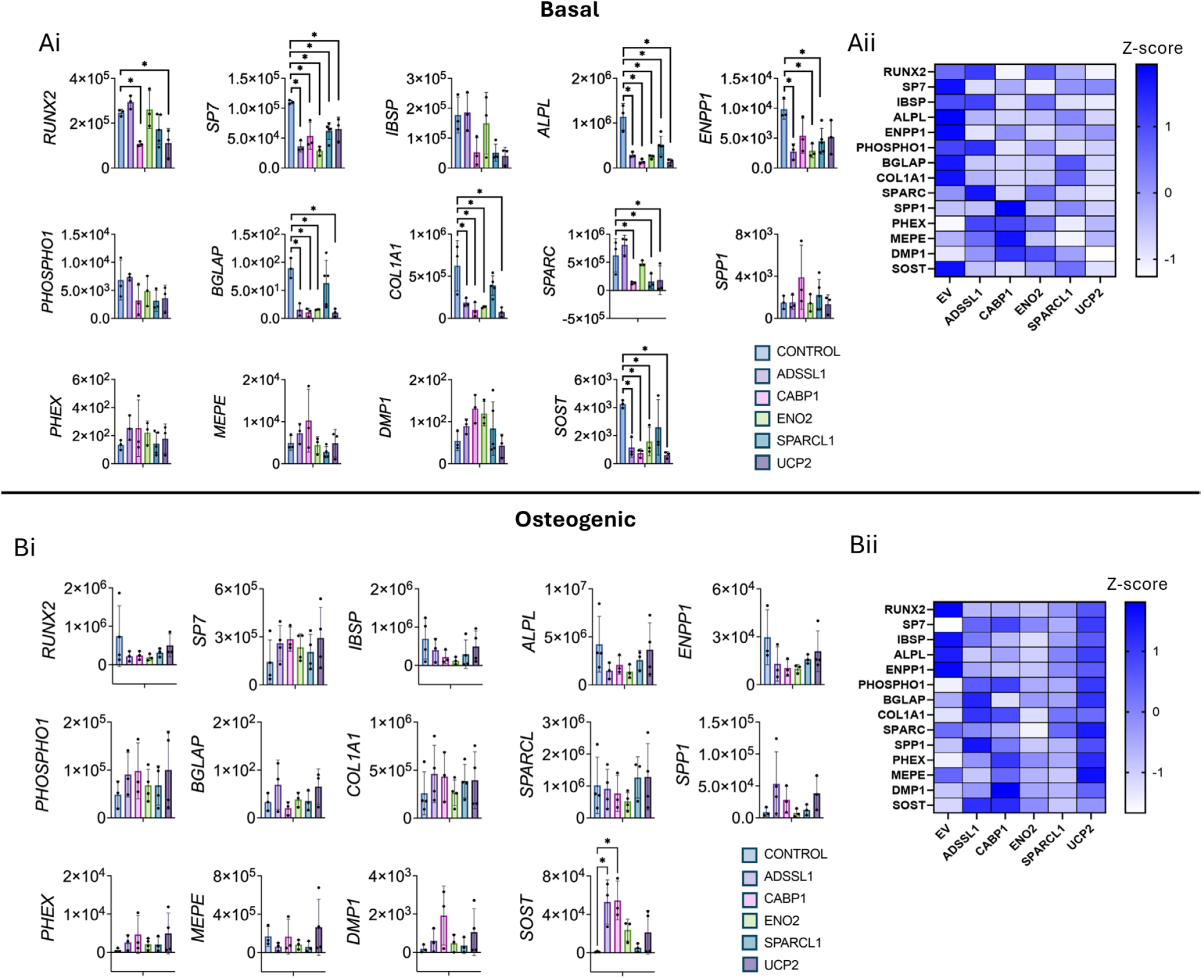
Overexpression of the genes of interest affects osteogenic gene expression. Expression of osteogenic marker genes in Saos2 cells overexpressing the genes of interest and cultured under basal conditions (Ai) and after 21 days of osteogenic culture (Bi). *ADSSL1* (purple bar), *CABP1* (Pink bar), *ENO2* (Green bar), *SPARCL1* (Teal bar) and *UCP2* (Dark purple bar). Blue bars represent the empty vector control (EV). Expression is shown relative to the *ACTB* housekeeping gene. Error bars represent the standard deviation from three to five experimental replicates. *p < 0.05. (Aii, Bii) Heatmaps show the mean expression levels of osteogenic marker genes using *Z*‐score normalisation. Blue indicates high expression, and white indicates low expression. The mean of three to five experimental replicates was used for these comparisons.

To determine if the gene modulation altered the expression profiles of the cells, we measured the expression of 14 osteogenic‐associated genes under both basal and osteogenic culture conditions. The overexpression of the candidate genes resulted in an overall trend to decrease the expression of osteogenic‐associated genes under basal cell culture. The early osteocyte marker *RUNX2* showed significant decreases when *CABP1* and *UCP2* were overexpressed. The early osteoblast marker *SP7* and the mature osteoblast marker *ALPL* showed significant decreases in expression following the overexpression of all genes of interest. *ENPP1* showed significant decreases in expression when *ADSSL1*, *ENO2* and *SPARCL1* were overexpressed. *BGLAP* and *COL1A1* were significantly decreased when *ADSSL1*, *CABP1*, *ENO2* and *UCP2* were overexpressed. *SPARC* was significantly downregulated following overexpression of *CABP1*, *SPARCL1* and *UCP2*. A significant decrease in osteocyte‐associated marker *SOST* was observed across all gene overexpression conditions, except when *SPARCL1* was overexpressed. In contrast, *IBSP*, *PHOSPHO1*, *SPP1*, *PHEX, MEPE* and *DMP1* showed no significant changes (Figure 5A).

However, following 21 days of osteogenic culture, the overexpression of the genes of interest resulted in few changes in osteogenic gene expression. The only significant change observed was an increase in *SOST* following the overexpression of *ADSSL1* and *CABP1* (Figure 5B).

### 3.5 Knockdown of Candidate Genes Results in Changes in the Expression of Osteogenic Genes Under Basal and Osteogenic Conditions

**FIGURE 6:**
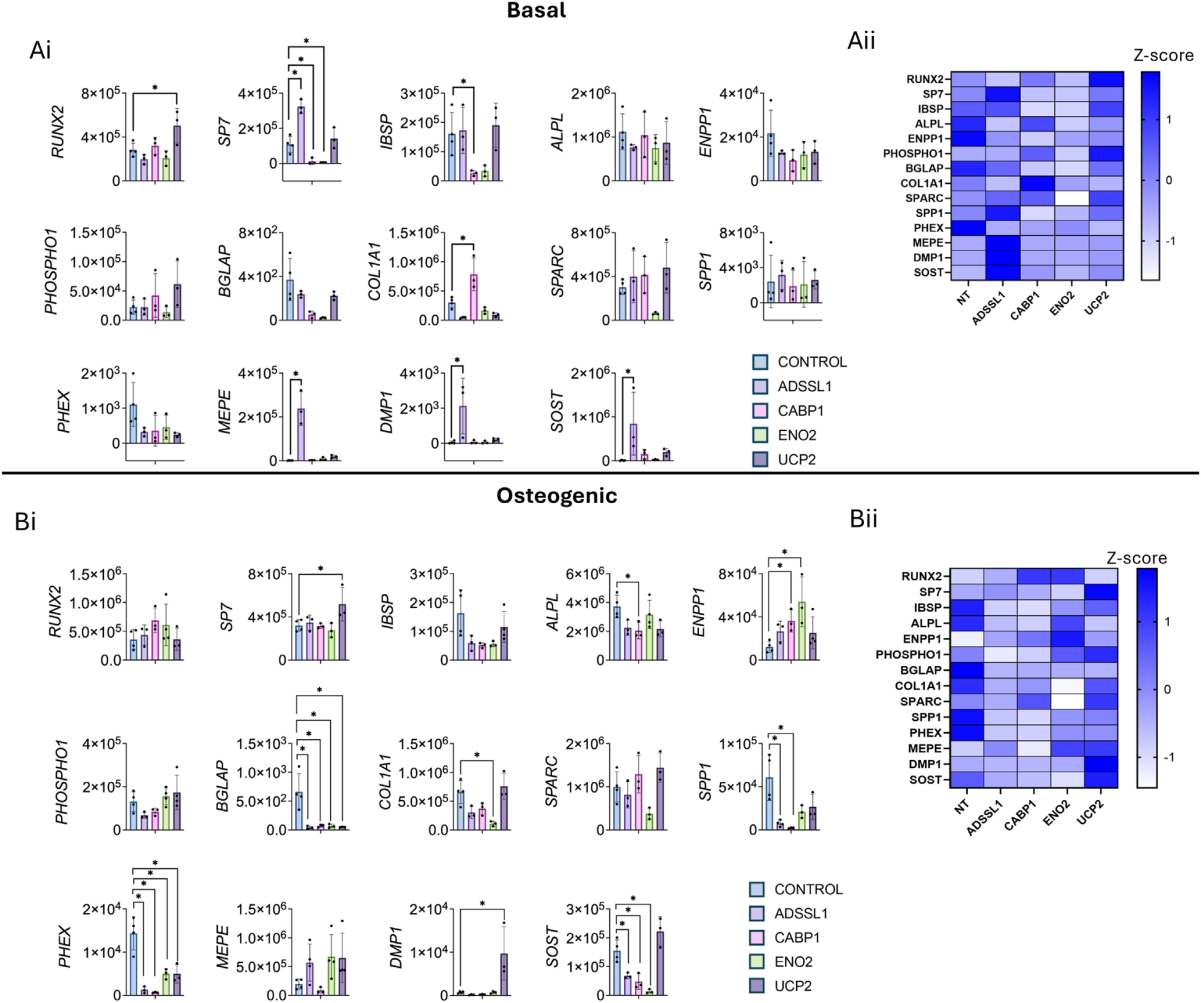
Knockdown of the genes of interest affects osteogenic gene expression. Expression of osteogenic marker genes in Saos2 cells following gene knockdown and cultured under basal conditions (Ai) and after 21 days of osteogenic culture (Bi). *ADSSL1* (purple bar), *CABP1* (Pink bar), *ENO2* (Green bar), *SPARCL1* (Teal bar) and *UCP2* (Dark purple bar). Blue bars represent the non‐target control (NT). Expression is shown relative to the *ACTB* housekeeping gene. Error bars represent the standard deviation from three to five experimental replicates. *p < 0.05. (Aii, Bii) Heatmaps show the mean expression levels of osteogenic marker genes using *Z*‐score normalisation. Blue indicates high expression, and white indicates low expression. The mean of three to four experimental replicates was used for these comparisons.

Knocking down the genes of interest resulted in fewer changes to osteogenic gene expression than their overexpression did under basal conditions. In basal conditions, knockdown of *UCP2* led to a significant increase in the early osteoblast marker *RUNX2*. Early osteoblast marker *SP7* showed a significant increase following *ADSSL1* knockdown but a significant decrease when *CABP1* or *ENO2* were knocked down. Knockdown of *CABP1* also resulted in a significant decrease of the osteoblast marker *IBSP* and in a significant increase in the mature osteoblast marker *COL1A1*. Additionally, *ADSSL1* knockdown caused a significant upregulation of the late osteoblast markers *MEPE* and *DMP1*, as well as the osteocyte marker *SOST*. In contrast, no significant changes were observed in the expression of mature osteoblast markers *ALPL*, *ENPP1*, *PHOSPHO1*, *BGLAP*, *SPARC* and *SPP1*, or the late osteoblast marker *PHEX*, following the knockdown of any of the genes (Figure 6A).

In contrast, under osteogenic conditions, knockdown of the genes of interest resulted in more changes to osteogenic gene expression than their overexpression did. The early osteoblast marker *SP7* was significantly increased when *UCP2* was knocked down. The mature osteoblast marker *ALPL* was significantly decreased following *CABP1* knockdown, while *ENPP1* showed a significant increase when either *CABP1* or *ENO2* was knocked down. *BGLAP* was significantly decreased following knockdown of all the genes of interest. Mature osteoblast marker *COL1A1* expression was significantly decreased when *ENO2* was knocked down, and *SPP1* was significantly decreased following knockdown of both *ADSSL1* and *CABP1*. The late osteoblast marker *PHEX* was significantly decreased when all four genes were knocked down. Late osteoblast marker *DMP1* showed a significant increased following *UCP2* knockdown, while the osteocyte‐associated marker *SOST* was significantly decreased when *ADSSL1*, *CABP1* or *ENO2* was knocked down. In contrast, no significant changes were observed in the expression of *RUNX2*, *IBSP*, *PHOSPHO1*, *SPARC* or *MEPE* following knockdown of any of the genes of interest (Figure 6B).

### 3.6 Bone Matrix Deposition and Mineralisation Are Affected by the Modulation of the Candidate Genes

**FIGURE 7:**
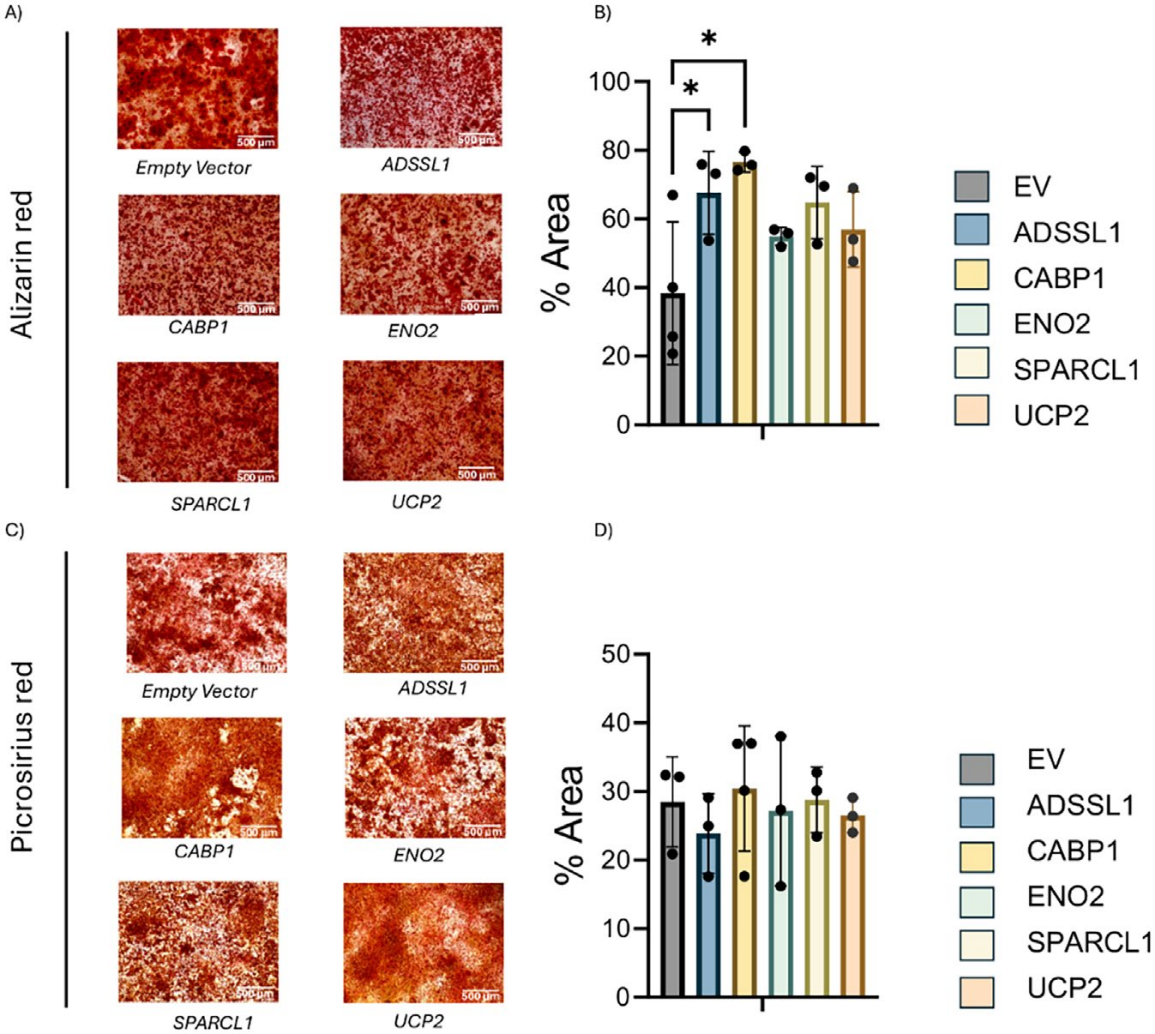
Overexpression of the genes of interest results in more diffuse mineralisation staining with no change in collagen staining. (A) Alizarin red staining for calcium deposits after 21 days of osteogenic culture in empty vector control cells (EV) and cells overexpressing the genes of interest. (B) Quantification of alizarin red staining showing the percentage area that is positively stained. (C) Picrosirius Red staining for collagen deposition after 21 days of osteogenic culture empty vector control cells (EV) and cells overexpressing the genes of interest. (D) Quantification of Picrosirius red staining showing the percentage area that is positively stained. Error bars represent the standard deviation from three experimental replicates. *p < 0.05. Scale bars = 500 μm.

To determine if the modulation of the genes of interest affected bone formation and mineralisation, we measured collagen and matrix deposition after 21 days of osteogenic culture. Robust levels of mineralisation (measured using only alizarin red due to the GFP marker gene in the overexpression construct) were observed after 21 days of osteogenic culture following the overexpression of all genes. There was an overall trend for the overexpression of the genes of interest to result in an increase in the percentage area stained compared to the empty vector control. These increases were significant following *ADSSL1* and *CABP1* overexpression. This appeared to be due to a more diffuse staining pattern following gene overexpression. Picrosirius Red staining for collagen matrix deposition revealed no significant changes following the overexpression of any of the genes (Figure 7).

**FIGURE 8:**
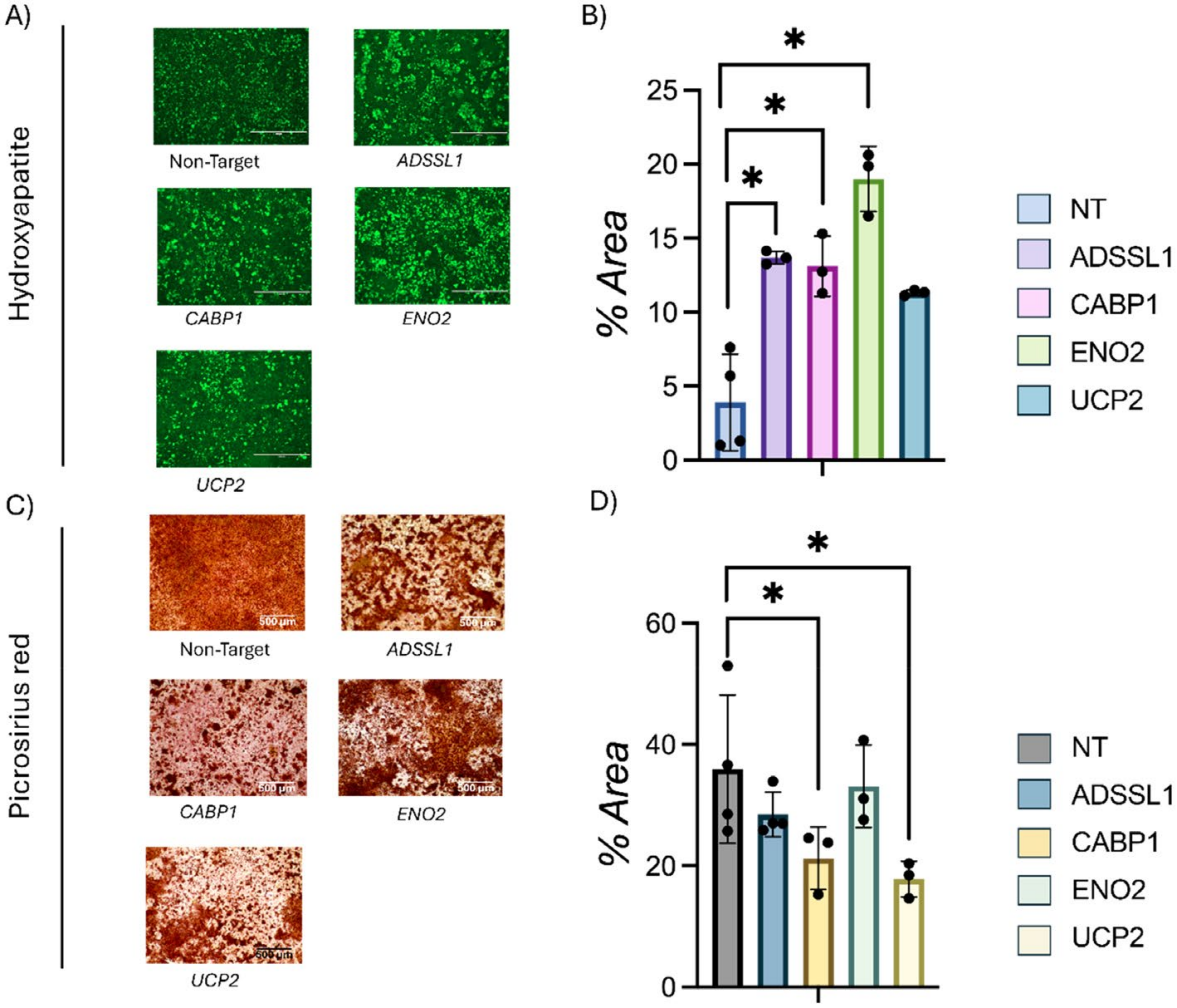
Knockdown of the genes of interest results in increased hydroxyapatite staining and a decrease in collagen staining. (A) Hydroxyapatite staining after 21 days of osteogenic culture in non‐target control (NT) cells and cells with knockdown of the genes of interest. (B) Quantification of hydroxyapatite staining showing the percentage area that is positively stained. (C) Picrosirius Red staining for collagen deposition after 21 days of osteogenic culture in non‐target control cells (NT) and cells with knockdown of the genes of interest. (D) Quantification of Picrosirius Red staining showing the percentage area that is positively stained. Error bars represent the standard deviation from three experimental replicates. *p < 0.05. Scale bars = 500 μm.

Following gene knockdown, matrix mineralisation was measured using hydroxyapatite staining after 21 days of osteogenic culture. There was a general trend for matrix mineralisation to increase following gene knockdown, which was significant for *ADSSL1*, *CABP1* and *ENO2*. Interestingly, Picrosirius Red staining for collagen matrix deposition resulted in a general trend to reduce the area stained, and this was significant when *CABP1* and *UCP2* were knocked down (Figure 8).

## 4 Discussion

This study aimed to determine if genes that were differentially expressed in iPSC‐osteoblasts derived from horses at high and low genetic risk of fracture had novel roles in bone formation. We first measured the expression of 42 of the differentially expressed genes in Saos2 cells cultured under basal (proliferative conditions) or after 21 days of osteogenic culture. Expression of 16 of the 42 genes was not detected. Only two of these genes (*GPHA4* and *MUC4*) were either not detected or not annotated in equine bone tissue in publicly available RNA sequencing datasets (Kuemmerle et al. 2016; Kemper et al. 2019). The discrepancy in expression may be because the genes are expressed by a bone cell type or stage that is not represented in our culture system. However, we could not access suitable positive control samples for our human primer sets and therefore cannot rule out that the lack of detection was due to the primers being used. The remaining 26 genes were all robustly expressed in Saos2 cells. Of these genes, *ABCG4*, *INHBE* and *PLCH2* were either not detected or not annotated in equine bone tissue in the available RNA sequencing datasets. However, *INHBE* was robustly expressed in cultured equine bone cells (Kuemmerle et al. 2016).

From these 26 genes, we then selected five genes (*ADSSL1*, *CABP1*, *ENO2*, *SPARCL1* and *UCP2*) for modulation studies. The selection of these genes is outlined in Figure 1B. We could successfully overexpress all five genes of interest, but it was not possible to knock down *SPARCL1* (SPARC‐like protein 1) in the Saos2 cells. Two shRNA constructs were tested, and in both cases, the antibiotic selection resulted in no surviving cells. This suggests that *SPARCL1* is an essential gene in these cells. *SPARCL1* is a paralog of *SPARC* (secreted protein acidic and cysteine rich [also known as osteonectin]) (Bertrand et al. 2013). SPARCL1 and SPARC are both secreted glycoproteins. However, while SPARC is known to have key roles in bone, regulating procollagen processing and collagen crosslinking (Rosset and Bradshaw 2016), the function of SPARCL1 in bone is yet to be elucidated. SPARCL1 has been implicated in cancer biology through its involvement in the regulation of cell adhesion, migration and proliferation and it may act as both an oncogene and tumour suppressor (Gagliardi et al. 2017). Therefore, it is not clear if *SPARCL1* is essential in Saos2 cells due to their cancerous origin, rather than their osteoblast‐like phenotype, and future work using alternative osteoblast cell lines may be required to confirm this. Overexpression of *SPARCL1* significantly reduced the viability of Saos2 cells cultured under basal conditions. It also resulted in significant reductions in *SP7*, *ALPL*, *ENPP1* and *SPARC* expression under basal conditions. However, no significant changes in gene expression, collagen deposition or matrix mineralisation were observed following *SPARCL1* overexpression after 21 days of osteogenic culture.

Unlike *SPARCL1*, the other genes studied (*ADSSL1*, *CABP1*, *ENO2*, *UCP2*) were all expressed at significantly lower levels in iPSC‐osteoblasts derived from horses at high genetic risk of fracture compared to those derived from horses at low genetic risk of fracture (Figure S1). For these genes, we were able to successfully overexpress them and knock them down in Saos2 cells. Overexpression of all the genes resulted in decreased cell viability and decreased osteogenic gene expression under basal conditions. However, following osteogenic differentiation there was little effect on osteogenic gene expression. We did not measure gene expression during the 21 days of osteogenic culture, and it is possible that there are temporal effects on gene overexpression. However, following osteogenic culture we also did not see substantial changes in mineralisation, although there was a trend for some modulations to result in a more diffuse staining pattern. Future work to quantify the effects of gene overexpression at earlier time points in the osteogenic culture would be beneficial.

In contrast, knockdown of the genes of interest produced relatively few effects on osteogenic gene expression under basal cell culture but following 21 days of osteogenic culture, gene knockdown consistently reduced collagen matrix deposition while increasing levels of hydroxyapatite staining suggesting that smaller amounts of matrix are deposited but undergo more mineralisation. There was not a clear correlation of this finding to gene expression: *COL1A1* was only significantly reduced following *ENO2* knockdown; genes associated with promoting mineralisation (*ALPL* and *PHOSPHO1*) did not increase; and genes associated with inhibiting mineralisation (*ENPP1*) increased. We consistently observed a decrease in *BGLAP* and *PHEX* expression following knockdown of each gene. BGLAP (also known as Osteocalcin) is highly expressed during mineralisation and binds to hydroxyapatite (Price et al. 1976; Hauschka and Wians 1989) where it may regulate crystal deposition and growth (Chen et al. 2015). *BGLAP* expression has been shown to increase during in vitro bone formation in MSCs (Nakamura et al. 2009; Tsao et al. 2017) and Saos2 cells (Ross et al. 2026) but *BGLAP* knockout mice studies have produced contrasting results depending on sex, age and genetic background (Ducy et al. 1996; Bailey et al. 2017; Berezovska et al. 2019; Diegel et al. 2020; Moriishi et al. 2020; Paracha et al. 2024). PHEX is a marker for osteocytes and is involved in matrix mineralisation (Robling and Bonewald 2020). The observed decreases in these genes therefore do not explain the increased matrix mineralisation. To further investigate this, performing temporal gene expression analysis during osteogenic culture, preferably using global transcriptomics analysis, would be required. By measuring global transcriptional changes (e.g., using RNA sequencing) in response to gene modulation, we could better determine what biological pathways and processes could lead to the observed changes in osteogenesis and identify if modulation of one gene also lead to changes in the expression of the other genes under investigation.

*ADSSL1* (Adenylosuccinate synthetase‐like 1) gene encodes an essential enzyme for initiating the purine synthesis pathway. This pathway is responsible for producing adenosine monophosphate (AMP) from inosine monophosphate (IMP) (Lipps and Krauss 1999). AMP is a key metabolite required for adenosine triphosphate (ATP) production. ATP is required for the production of inorganic pyrophosphate (PPi), which can inhibit mineralisation (Addison et al. 2007). Therefore, *ADSSL1* knockdown may result in lower ATP and PPi production which leads to the increased mineralisation observed. UCP2 (Uncoupling protein 2) belongs to the mitochondrial anion carrier protein family and is also involved in ATP production (Tian et al. 2018). Enolase 2 is involved in glycolysis (Zhou et al. 2024), which is required for osteoclast activity and osteoblast differentiation (Choi et al. 2024). Future work to measure ATP production, glycolysis and PPi synthesis following knockdown of these genes would help to elucidate the mechanisms behind the increased mineralisation observed. CABP1is a calcium binding protein. Many other calcium binding proteins have important roles in bone (e.g., BGLAP (Tsao et al. 2017) and SPARC) but it is not clear from our data how *CABP1* knockdown results in an increase in mineralisation and a deeper understanding of its location, binding partners and function in bone extracellular matrix is required.

We had previously demonstrated that LGALS1 knockdown in Saos2 cells results in increased matrix mineralisation (Ross et al. 2026). LGALS1 has already been described to be involved in osteoblast and osteoclast differentiation (Xu et al. 2021; Chen et al. 2022; Takeuchi et al. 2024) and was also expressed at lower levels in iPSC‐osteoblasts from high‐risk horses (Palomino Lago et al. 2025). It is interesting that in this study, knockdown of a further four genes with a similar reduced level of expression in iPSC‐osteoblasts from high‐risk horses also results in increased matrix mineralisation. Taken together, this suggests that genetic risk factors may affect the expression of genes whose roles converge to result in increased bone mineral density. In vivo studies on Thoroughbred horses with and without catastrophic distal limb fractures have provided mixed results on bone mineral density. Some studies have reported that bone mineral density increases with exercise but is not changed between fracture cases and controls (Bogers et al. 2016; Noordwijk et al. 2023). Reduced bone mineral density in subchondral lesions of focal osteopenia has been associated with proximal sesamoid bone fractures (Shaffer et al. 2021). Whereas other studies have reported increased bone mineral density in proximal sesamoid bone fractures (Shi et al. 2011) and in focal regions adjacent to the fracture site in lateral condylar fractures of the third metacarpal (Loughridge et al. 2017). These differences may reflect limitations in technology to detect focal or subtle changes and/or confounding environmental factors.

This study had some limitations as we did not confirm overexpression and knockdown at the protein levels. With the exception of *SPARCL1*, we did not perform gene knockdown using multiple shRNAs and we did not perform rescue experiments of the knockdowns to help validate our findings and demonstrate that they were not due to off‐target effects. Furthermore, this study does not replicate the complexities of the in vivo situation and the degree of knockdown achieved also did not precisely match the differences observed in expression in the original study between iPSC‐osteoblasts derived from genetically high‐risk and low‐risk horses (Palomino Lago et al. 2025; Table S2). We also utilised a human cell line as an equine osteoblast cell line was not available at the time. We have since derived equine immortalised osteoblasts (Palomino Lago et al. 2026) which can be utilised in future studies.

In summary, our results demonstrate that *ADSSL1*, *CABP1*, *ENO2* and *UCP2*, which are all expressed at significantly lower levels in iPSC‐osteoblasts derived from genetically high‐risk horses, have novel roles in bone formation with their knockdown promoting mineralisation. Further investigation into the other fracture‐associated genes that have been identified in Thoroughbred racehorses (Palomino Lago et al. 2025) is therefore warranted to determine if matrix deposition and mineralisation are key pathways underpinning the genetic risk of fracture.

## Author Contributions

**Amy C. Ross:** methodology, investigation, writing – original draft, writing – review and editing, visualization, validation, data curation, formal analysis. **Ellison S. Lumsden:** investigation, writing – review and editing, formal analysis, validation, data curation, methodology. **Caroline Flood:** investigation, formal analysis, writing – review and editing, data curation. **Jayesh Dudhia:** writing – review and editing, supervision. **Androniki Psifidi:** supervision, writing – review and editing. **Deborah J. Guest:** writing – review and editing, writing – original draft, supervision, resources, project administration, conceptualization, methodology, funding acquisition, visualization.

## Funding

This work was supported by Alborada Trust.

## Conflicts of Interest

The authors are affiliated with The Royal Veterinary College, which holds patent WO 2015/019097 “Predictive Method for Bone Fracture Risk in Horses” in relation to this work. This patent claims a method of predicting fracture risk in horses using one or more genetic variations from within the associated region on ECA18.

## Supporting information


**Figure S1:** Expression of the genes of interest in iPSC‐osteoblasts derived from horses at genetically low risk (blue bars) and high risk (red bars) of catastrophic fracture. Expression is shown as normalised count (data taken from Palomino Lago et al. 2025).
**Table S1:** Primers used in qPCRs.
**Table S2:** Comparison of the expression of the genes in high‐risk (HR) versus low‐risk (LR) iPSC osteoblasts (data adapted from Palomino Lago et al. 2025) and the knockdown levels achieved under basal and osteogenic culture. N/A = not applicable as knockdown of *SPARCL1* resulted in cell death.

## Data Availability

All data generated and analysed in this study are included in the published article and the Supporting Information.
