# Supplementary material for: Genes Associated With Fracture Risk in Thoroughbred Horses Have Novel Roles in Osteogenesis
Source: Anim Genet. 2026 Jul 8;57(4):e70166. doi: 10.1002/age.70166 (PMC13344464; doi:10.1002/age.70166)
Supplement: Supplementary file 1 — Figure S1: Expression of the genes of interest in iPSC‐osteoblasts derived from horses at genetically low risk (blue bars) and high risk (red bars) of catastrophic fracture. Expression is shown as normalised count (data taken from Palomino Lago et al. 2025). Table S1: Primers used in qPCRs. Table S2: Comparison of the expression of the genes in high‐risk (HR) versus low‐risk (LR) iPSC osteoblasts (data adapted from Palomino Lago et al. 2025) and the knockdown levels achieved under basal and osteogenic culture. N/A = not applicable as knockdown of SPARCL1 resulted in cell death. [file AGE-57-0-s001.docx]

**Supplementary Table 1.** Primers used in qPCRs

| Gene | Protein Name | Gene ID | Forward Primer (5’ – 3’) | Reverse Primer (5’ – 3’) |
| --- | --- | --- | --- | --- |
| *ACTB* | Actin Beta | 60 | GCCTCGCCTTTGCCGAT | CGCGGCGATATCATCATCC |
| *ABCG4* | ATP binding cassette subfamily G member 4 | 64137 | AGGTGGAGAACCACATCACT | AGAGGCACTTGAGAAGGGTC |
| *ALPL* | Alkaline Phosphatase | 249 | CACGGAACTCCTGACCCTTG | TCCTGTTCAGCTCGTACTGC |
| *ADSSL1* | Adenylosuccinate synthase 1 | 122622 | AGAAAGGCCTGAAGGACTGG | CCCTTCTTGGTGGTGCCTAT |
| *AMPD3* | Adenosine monophosphate deaminase 3 | 272 | CGACCTCACTGTGGACTCAC | ATACAGGTCACGCAGCTCAC |
| *ASNS* | Asparagine synthetase | 40 | GATCGAACTACTGCTGCCCA | TTTCTGGTGGCAGAGACAAGT |
| *BGLAP* | Bone gamma-carboxyglutamate protein (Osteocalcin) | 632 | CACTCCTCGCCCTATTGGC | CCCTCCTGCTTGGACACAAAG |
| *CABP1* | Calcium binding protein 1 | 9478 | AGATCACTGCGACCAGAGGA | CAGATCCCGGCAGTTGATGT |
| *CLHC1* | Clathrin heavy chain linker domain containing 1 | 130162 | GCAGGGAGACACCTTCCATC | TCTTCAAGGAGCGTCACCAC |
| *COL1A1* | Collagen Type 1 Alpha 1 | 1277 | GCCCTGCTGGTGCTCG | GGACCTTCAGAGCCTCGGG |
| *COTL1* | Coactosin like F-actin binding protein 1 | 23406 | TCCCTACTAAGGCCCAGACC | CCTTCTCACCACCGAGCAAT |
| *DIPK2A/C16H3orf58* | Divergent protein kinase domain 2A | 205428 | TGAGTGTGCCAACCCAAAGA | CACGCAGTTCTTTTGCAGCC |
| *DMP1* | Dentin Matrix Acidic Phosphoprotein 1 | 1758 | AGTGGCTTCATTGGGCATAGA | TCCCCAAAGGAACATGAGCAG |
| *ENO2* | Enolase 2 | 2026 | TCCCACTGATCCTTCCCGAT | TCCCACTGATCCTTCCCGAT |
| *ENPP1* | Ectonucleotide pyrophosphatase/phosphodiesterase 1 | 5167 | CCCTCAGTGGCAACTTGCAT | GCCAACAAAGAGGGCTTGC |
| *FRMD4A* | FERM domain containing 4A | 55691 | GGAAGTTCATGACCCACGCA | ATGCCGGACATGCATACCAC |
| *IBSP* | Integrin Binding Sialoprotein | 3381 | AGGACTGCCAGAGGAAGCAA | GAGAAAGCACAGGCCATTCC |
| *INHBE* | Inhibin subunit beta E | 83729 | TCCTCAAGTGAGGGGAGGAG | CCTCAACAGCCCTTACCCTG |
| *KIZ* | Kizuna centrosomal protein | 55857 | ACGCACCAACAAGAGAACCT | CCCTCGGTACGTCTGAGTCT |
| *MEPE* | Matrix Extracellular Phosphoglycoprotein | 569,55 | TCCTTTTCAGTGTGACCTGGG | TCCACACAGCTTTGCTTAGT |
| *NAP1L5* | Nucleosome assembly protein 1 like 5 | 266812 | AAAAGCTGCAGAAGCGATGC | GCTCTTGGATCTTGGCGAGT |
| *NXPH4* | Neurexophilin 4 | 11247 | CCGGAATGGTTCCTCTTGCT | TCCGGACTCTGGTATCTGGG |
| *PEG10* | Paternally expressed 10 | 23089 | CCATCCTTCCTGTCTTCGCA | TCCCAGCTGTAGCTTCACTTC |
| *PHEX* | Phosphate Regulating endopeptidase X-Linked | 5251 | ACCTTAGCAGGCGCTTTCAG | TTTGTCCCATTGAGGCAGCA |
| *PHOSPHO1* | Phosphoethanolamine/phosphocholine phosphatase 1 | 162466 | GTAAGCACCCCTTGCTCCAT | GTCACACGTTCGTGGGGG |
| *PLCH2* | Phospholipase C eta 2 | 9651 | AGCATGATGCCAGGCTACAG | CTGCTTGACCTTACCGCTGA |
| *PLPPR3* | Phospholipid phosphatase related 3 | 79948 | CAAGATCCCGAAGGACAGCA | TCATAGCACTGGAAGCCCAC |
| *PPP1R2* | Protein phosphatase 1 regulatory inhibitor subunit 2 | 5504 | AGACTGCAGATGGAGAAAGCA | TGGTCACTTGGAGTAGATCCTTG |
| *PTK7* | Protein tyrosine kinase 7 | 5754 | CTGCAGTGGCTCTTTGAGGA | AGCGGTAGATCCCTGCATTG |
| *RUNX2* | RUNX Family Transcription Factor 2 | 860 | ACCGAGACCAACAGAGTCATTTA | GTCACTGTGCTGAAGAGGCT |
| *SCRN2* | Secernin 2 | 90597 | GGTATCCCCCAGGGAGTAGG | GGAGTGGGGGAGGTCTATCA |
| *SOST* | Sclerostin | 509,64 | CACCACCCCTTTGAGACCAA | GTCACGTAGCGGGTGAAGT |
| *SPARC* | Secreted Protein Acidic And Cysteine Rich | 6678 | CAAGAAGCCCTGCCTGATGA | TCTTCGGTTTCCTCTGCACC |
| *SPARCL1* | SPARC like 1 | 8404 | GCTGCAATCCCGACAAATGC | AGTGTTGTCAGGTGCTACCG |
| *SPP1* | Secreted phosphoprotein 1(Osteopontin) | 6696 | AGCAGAATCTCCTAGCCCCA | TGGTCATGGCTTTCGTTGGA |
| *SP7* | Osterix | 121,340 | GCCATTCTGGGCTTGGGTAT | TGCAGGTATCAGGCACAAGG |
| *TDRKH* | Tudor and KH domaining containing | 11022 | CACTCGACGGCTGACTGG | GGCCCAGGGCTATTTTCTGA |
| *TMEM38A* | Transmembrane protein 38A | 79041 | ATTGCAACTGGGTGGGTCAA | TGTGCAGGATCTCGTTGGTC |
| *UCP2* | Uncoupling protein 2 | 7351 | CCTCTCCCAATGTTGCTCGT | GGCAAGGGAGGTCATCTGTC |
| *WNK4* | WNK lysine deficient protein kinase 4 | 65266 | TGGCATCATGCGAAGGAACT | CTGTTCTGAATTCACATCCTGC |
| *ZSWIM5* | Zinc finger SWIM-type containing 5 | 57643 | GCCTCATGATCCAGACCTGT | GCGTGAACCAGCGAGGATAA |

**Supplementary Table 2.** Comparison of the expression of the genes in high-risk (HR) versus low-risk (LR) iPSC osteoblasts (data adapted from (Palomino Lago *et al.* 2025) and the knockdown levels achieved under basal and osteogenic culture. N/A = not applicable as knockdown of *SPARCL1* resulted in cell death.

| **Gene** | **HR v LR iPSC-osteoblasts** | **KD basal conditions** | **KD osteogenic conditions** |
| --- | --- | --- | --- |
| *ADSSL1* | 5-fold lower in HR | 3.5-fold reduction | 2.1-fold reduction |
| *CABP1* | 42-fold lower in HR | 1.25-fold reduction | 2.0-fold reduction |
| *ENO2* | 6-fold lower in HR | 3.63-fold reduction | 41.4-fold reduction |
| *UCP2* | 4-fold lower in HR | 9.4-fold reduction | 442.5-fold reduction |
| *SPARCL1* | 522-fold **higher** in HR | N/A | N/A |


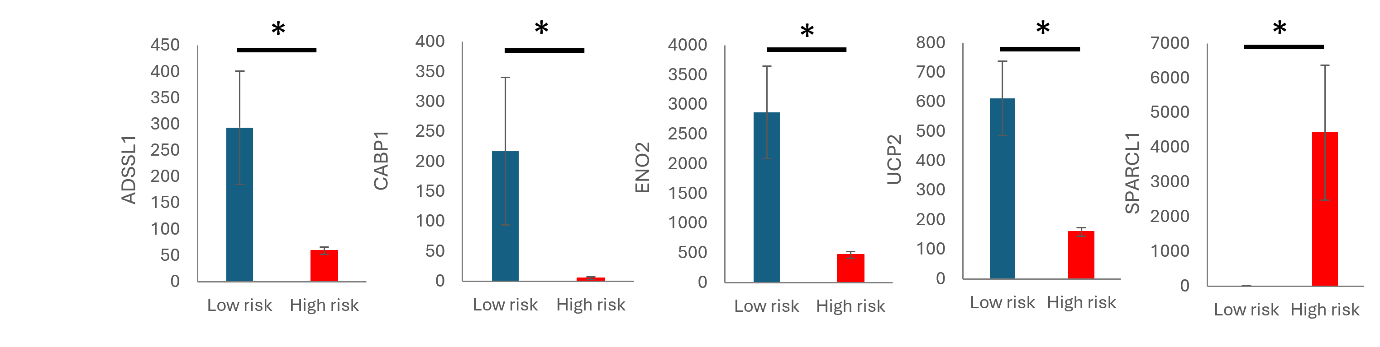


**Supplementary Figure 1.** Expression of the genes of interest in iPSC-osteoblasts derived from horses at genetically low risk (blue bars) and high risk (red bars) of catastrophic fracture. Expression is shown as normalised count data taken from (Palomino Lago *et al.* 2025).
